# The influence of nasal microbiome diversity and inflammatory patterns on the prognosis of nasal polyps

**DOI:** 10.1038/s41598-021-85292-5

**Published:** 2021-03-18

**Authors:** Weigang Gan, Hongting Zhang, Fengjuan Yang, Shixi Liu, Feng Liu, Juan Meng

**Affiliations:** Department of Otorhinolaryngology–Head and Neck Surgery, West China Hospital, West China School of Medicine, Sichuan University, Chengdu, Sichuan People’s Republic of China

**Keywords:** Clinical trial design, Applied microbiology, Microbial communities

## Abstract

To understand the inflammatory microenvironment and microbiome factors for prognosis of chronic rhinosinusitis with polyps (CRSwNP), we explored the difference in characteristics of the microbiome of the nasal sinuses and inflammatory cytokines between recurrent and non-recurrent groups. We collected nasal secretions and polyp tissue from 77 CRSwNP patients. Then, we extracted microbial DNA from cotton swabs, performed high-throughput sequencing based on 16S rRNA to detect bacterial community composition, and analyzed cytokines such as IL-5, IL-8, IL-17a, IL-17e, IL-18, IL-27 and INF-gamma from polyp tissue using Luminex. The eosinophil and neutrophil cells in the peripheral blood and polyp tissue were counted. Postoperative follow-up of patients with CRSwNP for 1 year was conducted to record the recurrence of nasal polyps and analyze the correlation between the recurrence of nasal polyps and the characteristics of inflammatory cytokines, inflammatory cell count and nasal microbial diversity. After 1 year of follow-up, there were 12 recurrent patients, including 5 males and 7 females. Postoperative recurrence of nasal polyps was not significantly correlated with age, sex, asthma, allergic rhinitis or other allergic diseases in CRSwNP patients. In terms of the total nasal symptom score, the recurrent group was significantly higher than the non-recurrent group. In nasal polyp tissues, eosinophils (40.83/HP) and neutrophils (30.83/HP) in patients with CRSwNP in the recurrent group were significantly higher than those in the non-recurrent group (13.72/HP), and neutrophils (18.5/HP) were also significantly higher in the recurrent group than the non-recurrent group. The expression levels of IFN-, IL-17A, IL-17E and IL-18 were significantly higher in the recurrent group than in the non-recurrent group, and the positive rates were not different. In Southwest China, *Enterobacteria* and anaerobic bacteria may be correlated with the inflammatory pattern expression of nasal polyps. The neutrophil-mediated inflammatory response plays an important role in patients with CRSwNP in Southwest China and is correlated with nasal polyp recurrence. Recurrence of nasal polyps after endoscopic sinus surgery may be potentially associated with a reduced abundance of protective microorganisms and an increased number of pathogenic microorganisms.

## Introduction

The incidence of chronic rhinosinusitis with nasal polyps (CRSwNP) is increasing yearly, and this condition is a primary cause of morbidity and medical expenditure in European and American countries, which makes it of research value from the perspective of both epidemiology and health economics^1^. Nasal polyps treatment has been developed as a comprehensive approach including surgery, drugs, biological treatment and other means, but the recurrence rate of nasal polyps or sinusitis after nasal endoscopy is still high. Some scholars have reported recurrence rates reaching 80% and secondary surgery rates reaching 42%, and some have reported 57% and 46%, respectively^2^. Therefore, increasing attention has been given to the factors affecting the recovery of nasal polyps after surgery, among which the effects of the nasal microenvironment and immunophenotype on nasal polyps are particularly prominent.

With the deepening of the research, people have gradually realized that the nasal microbes for nasal inflammatory diseases (including nasal polyps) have a certain impact, regardless of whether the disease is in the early stage. In vitro research helps explore nasal sinus inflammatory disease pathogens, and modern molecular biology techniques provide 16S ribosomal RNA(rRNA) sequencing data and reliable means of studying the nasal cavity flora; however the latter approach is more accurate in reflection of the nasal cavity flora changes in nasal sinus inflammatory disease. High-throughput sequencing technology showed that changes in the core microbiome occurred in patients with chronic sinusitis compared to that in control subjects^3–5^. The inflammatory microenvironment was another key point in CRSwNP patients that we addressed. Based on previous studies related to the inflammatory microenvironment of nasal polyp tissues^6,7^, seven factors, IL-5, IL-8, IL-17A, IL-17E, IL-18, IL-27 and IFN-γ, were selected for analysis. We selected inflammatory cytokines as markers referring to the four types of inflammation in nasal polyp tissues. Patients with CRSwNP were divided into different types according to inflammatory patterns and the characteristics of their respective nasal microbiome. These results provide a basis for recognizing the microbiome diversity of patients with different inflammatory patterns of CRSwNP and exploring the effects of nasal microbial diversity and inflammatory types on the prognosis of nasal polyps.

## Results

### Comparison of microbiome composition between CRSwNP patients and the control subjects

**Figure 1:**
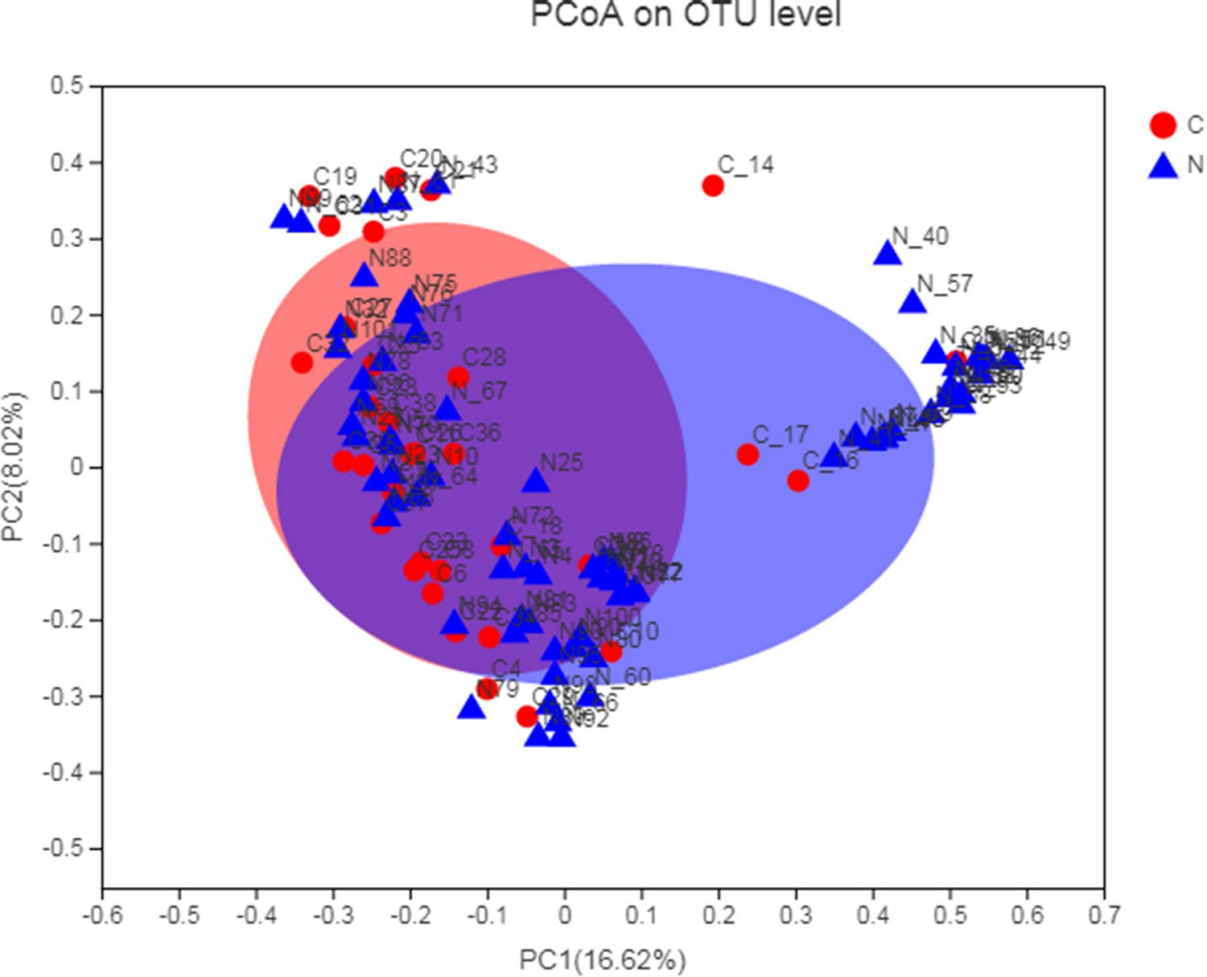
A principal coordinates analysis (PCoA) of bacterial communities was calculated with non-constrained data dimensionality reduction analysis method. The plots’ central tendency indicates the interpersonal variability of the two groups.

**Figure 2:**
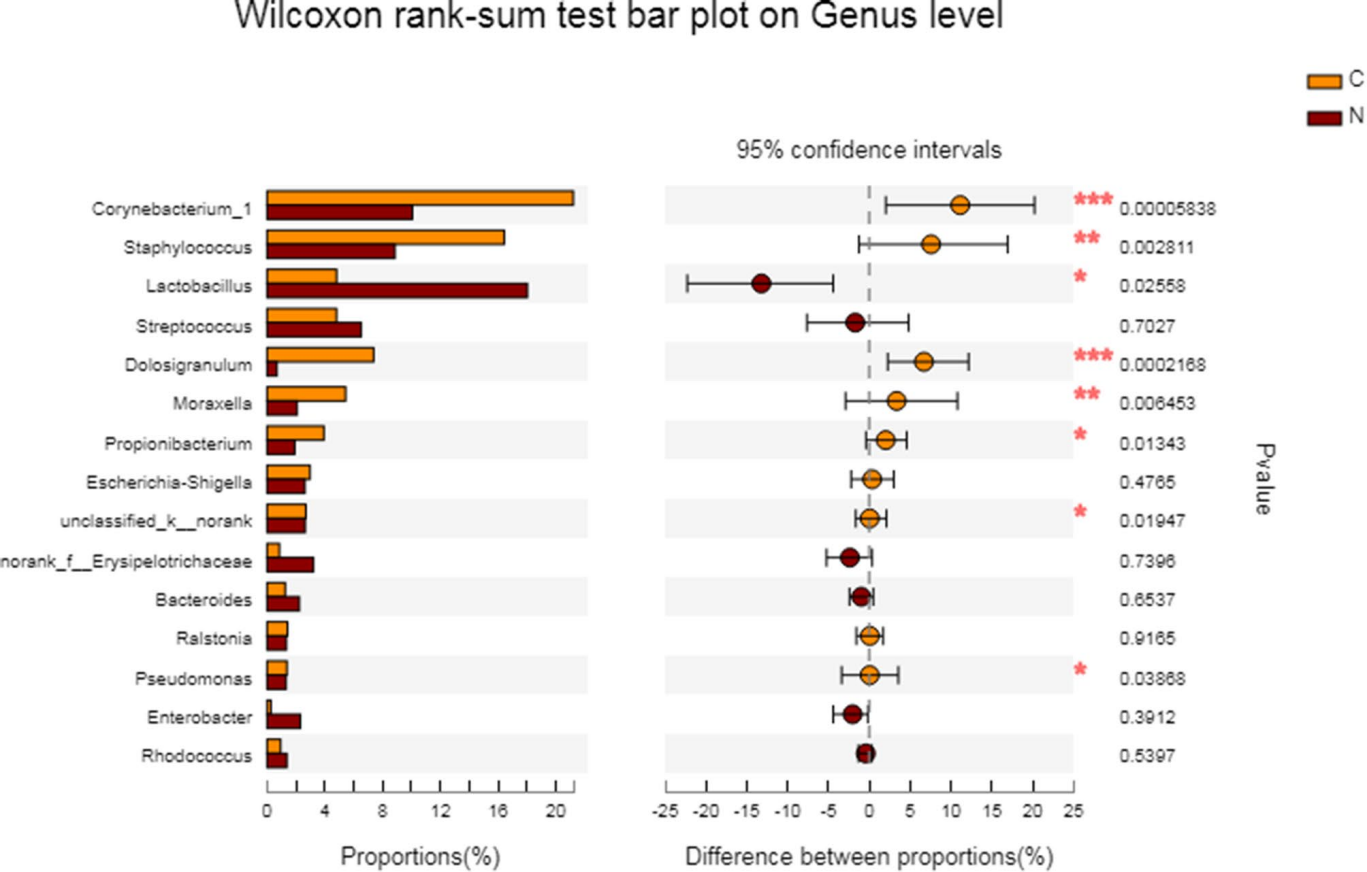
The Wilcoxon rank sum test was used to compare the difference in nasal microbial colony composition between recurrent and non-recurrent nasal polyp patients at the general level. *Note*: The right side of the figure shows the *P* value before FDR correction, *0.01 < *P* ≤ 0.05, **0.001 < *P* ≤ 0.01, ***P ≤ 0.001. After correction by FDR, only *Corynebacterium* had a PDR *P* = 0.047, and *Dolosigranulum* had PDR *P* = 0.012; the difference was statistically significant.

Principal coordinates analysis (PCoA) plots reflect the beta diversity of the microbial community among groups. In our study, we observed that between two groups most microbial distributions are overlapping and only a minority of samples are specific to each group (Fig. 1). Wilcoxon rank sum test was used to compare the nasal microbial diversity of CRSwNP patients and the control subjects. At the phylum level, the relative abundance of *Actinobacteria* was significantly lower in the CRSwNP group (16.07%) than that in the control group (31.42%) (FDR *P* = 0.041). At the genus level, the relative abundance of *Corynebacterium* in the CRSwNP group was 10.07%, which was significantly lower than that in the non-CRS group (21.21%) (FDR *P* = 0.047). The relative abundance of *Dolosigranulum* in the CRSwNP group was 0.69%, which was significantly lower than that in the non-CRS group (7.38%) (FDR *P* = 0.012) (Fig. 2).

### Clinical data of CRSwNP patients in the recurrent and non-recurrent groups

For the 77 patients with CRSwNP who underwent endoscopic sinus surgery (ESS), we conducted a 1-year follow-up and found 12 recurrent patients, including 5 males and 7 females. In the recurrent group, there were 3 CRSwNP patients with asthma, 4 with allergic rhinitis, and 0 with eczema; however, in the non-recurrent group, there were 8 with asthma, 10 with allergic rhinitis, and 6 with eczema. The epidemiological data of the two groups of patients are listed in Table 1. According to the data, there was no difference between the two groups in age, gender, asthma, allergic rhinitis or other allergic diseases. The Lund-Mackay scores and total nasal symptom scores (TNSS) of the recurrent group was significantly higher than that of the non-recurrent group (*P* = 0.001, *P* = 0.000).

**Table 1:** Comparison of clinical characteristics of CRSwNP patients in the recurrent and non-recurrent groups.

	Recurrent	Non-recurrent	Statistics	*P* value
Amount (proportion)	12(15.6%)	65(84.4%)		
Male/female	5/7	29/36	x^2^ = 0.036	0.85^a^
Age	48.62 ± 6.33	49.73 ± 9.16	F = 0.605	0.437^b^
Asthma (amount, %)	3(14)	8(6)	x^2^ = 1.768	0.184^a^
AR (amount, %)	4(18)	10(17)	x^2^ = 2.194	0.139^a^
Eczema (amount, %)	0(8)	6(8)	x^2^ = 1.201	0.273^a^
Bilateral CT score (Lund-Mackay)	22.50(14.00–24.00)	10.50(5.25–19.75)	Z = 3.435	0.001^c^
VAS (TNSS)	42.3(30.2–67.1)	37.76(29.38–50.3)	Z = − 4.93	0.000^c^

^a^Chi-square test, ^b^single factor analysis of variance, ^c^Mann-Whitney U test, *P* < 0.05.

### Comparison of inflammatory mediators between the recurrent group and the non-recurrent group

In terms of peripheral blood inflammatory cell count, the neutrophil count (median 3.82 × 109/L) in the recurrent group was higher than that in the non-recurrent group (median 3.59 × 109/L). There was no significant difference in peripheral blood eosinophil count between the two groups. In the nasal polyp tissues, the number of eosinophils (40.83/HP) and neutrophils (30.83/HP) in the recurrent group was higher than that in the non-recurrent group (13.72/HP), and the differences were statistically significant. Regarding the expression level of inflammatory mediators, 2 patients with positive IL-5 and 10 patients with negative IL-5 in the recurrent group were compared, and the difference was not statistically significant (X^2^ = 0.242, *P* = 0.623). The median expression of IL-8 in the recurrent group was 3,152.25 pg/ml and that in the non-recurrent group was 544.95 pg/ml. The difference was statistically significant, that is, the recurrent group was significantly higher than the non-recurrent group. The expression levels of IFN-, IL-17A, IL-17E and IL-18 were significantly higher in the recurrent group than in the non-recurrent group (IFN- (61.75 pg/ml in the recurrent group, 26.97 pg/ml in the non-recurrent group), IL-17A (15.27 pg/ml in the recurrent group, 6.77 pg/ml in the non-recurrent group), IL-17E (223.38 pg/ml in the recurrent group, 56.62 pg/ml in the non-recurrent group) and IL-18 (30.83 pg/ml in the recurrent group, 19.07 pg/ml in the non-recurrent group)). However, from the perspective of expression level, there was no significant difference in the positive rates of the four inflammatory factors between the recurrent group and the non-recurrent group. There was no significant difference in the positive rate or median expression of IL-27 between the recurrent and non-recurrent groups (Table 2).

**Table 2:** Comparison of inflammatory mediators between recurrent and non-recurrent CRSwNP patients.

	Non-recurrent	Recurrent	Statistic	*P* value
Peripheral blood eosinophil count (× 10^9^/L)	0.31(0.05–0.25)	0.40(0.22–0.60)	Z = − 1.837	0.066
Peripheral blood neutrophil count (× 10^9^/L)	3.82(2.99–5.37)	3.59(2.79–4.05)	Z = − 2.226	0.026^a^
Eosinophil count in polyp tissue (/HP)	40.83(22.33–102.0)	13.72(13.5–48.33)	Z = − 6.997	0.000^a^
Neutrophils count in polyp tissue (/HP)	30.83(20.33–56.44)	18.5(12.0–26.08)	Z = − 8.243	0.000^a^
**IL-5**				
Negative Positive	10 2	50 15	x^2^ = 0.242	0.623
**IL-8 (pg/ml)**				
Negative Positive	3152.25 (1627.0–5144.94)	544.95 (520.72–1889.53)	Z = − 17.885	0.000^a^
**IFN-γ (pg/ml)**				
Negative Positive	61.75(30.11–90.47)	26.97(23.83–41.14)	Z = − 3.424	0.001^a^
**IFN-γ**				
Negative Positive	2 10	10 55	x^2^ = 0.013	0.91
IL-17A (pg/ml)	15.27(6.77–20.2)	6.77(1.26–11.93)	Z = − 5.27	0.000^a^
**IL-17A**				
Negative Positive	3 9	16 49	x^2^ = 0.001	0.977
**IL-17E (pg/ml)**				
Negative Positive	223.38 (167.87–872.74)	56.62 (56.62–219.29)	Z = − 21.04	0.000^a^
**IL-17E**				
Negative Positive	4 8	15 50	x^2^ = 0.573	0.449
IL-18 (pg/ml)	30.83(20.33–56.44)	19.07(12.0–26.08)	Z = − 8.243	0.000^a^
**IL-18**				
Negative Positive	2 10	2 63	x^2^ = 3.799	0.051
**IL-27 (pg/ml)**				
Negative Positive	138.46 (86.11–248.12)	98.99 (60.86–178.94)	Z = − 0.941	0.347
**IL-27**				
Negative Positive	4 8	20 45	x^2^ = 0.031	0.86

^a^*P* < 0.05.

### Comparison of microbiome composition between the recurrent group and the non-recurrent group

**Figure 3:**
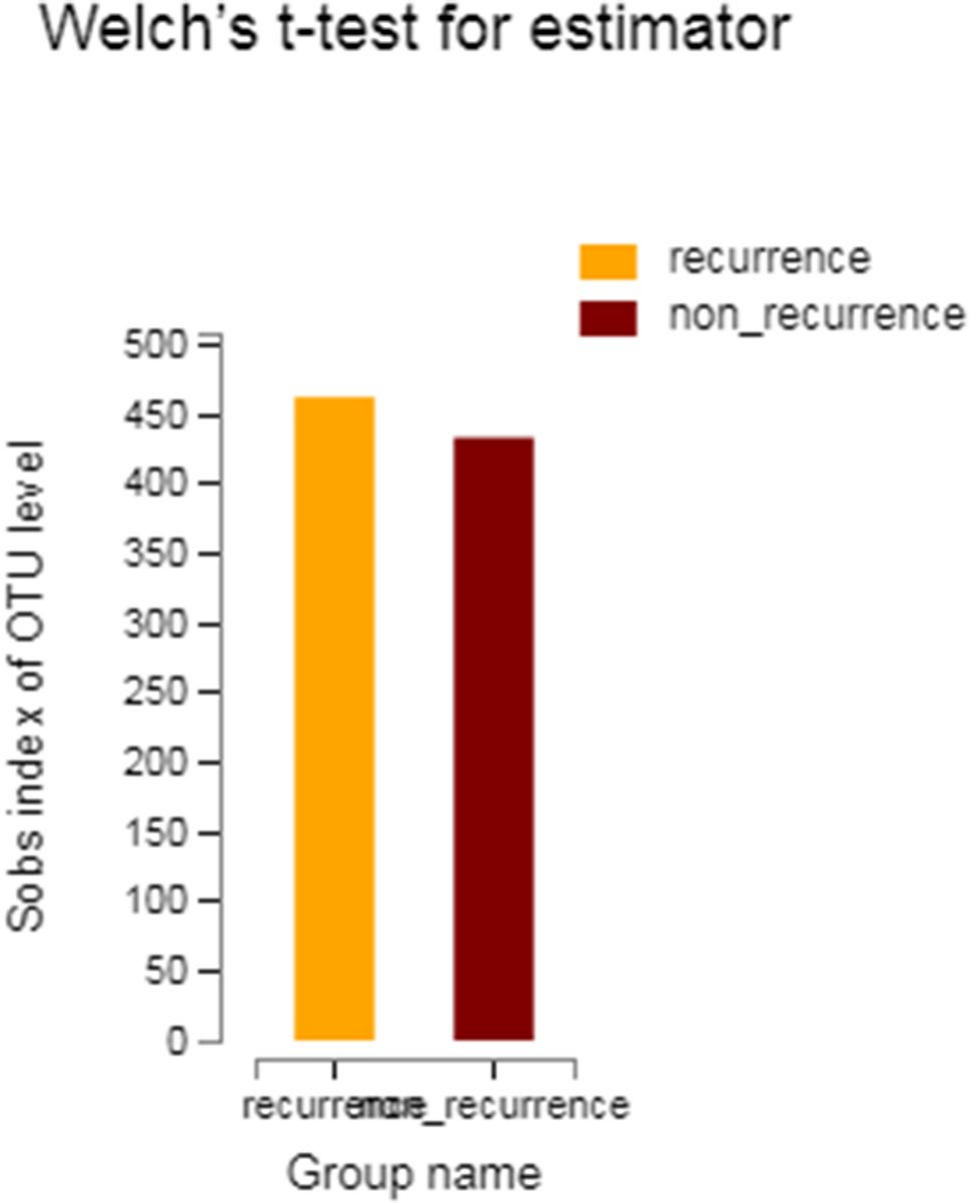
Differences in the Sobs index at the OTU level between the postoperative recurrent group and the non-recurrent group.

The mean microbial richness was compared between the recurrent group and the non-recurrent group. The results showed that there was no statistically significant difference in the Sobs index between the recurrent group (462.75) and the non-recurrent group (433.66) (*P* = 0.852), indicating that there was no significant difference in nasal microbiome richness between the two groups (Fig. 3).

For patients with CRSwNP in the recurrent group and the non-recurrent group, the most abundant bacterial categories were relatively concentrated in the following groups, including *Firmicutes* (non-recurrent group 49.19% vs recurrent group 52.99%), *Proteobacteria* (non-recurrent group 17.33% vs recurrent group 24.58%), *Actinobacteria* (non-recurrent group 18.0% vs recurrent group 2.626%), *Bacteroidetes* (non-recurrent group 8.31% vs recurrent group 14.65%), *Fusobacteria* (1.79% in non-recurrent group, 0.279% in recurrent group) and *Spirochaetae* (0.178% in non-recurrent group, 1.599% in recurrent group) (Table 3).

**Table 3:** Dominant groups and their average relative abundance in the recurrent and non-recurrent groups.

Taxonomic level	Non-recurrent (%)	Recurrent (%)	*P* value	FDR *P* value
**Phylum level**				
*Firmicutes*	49.19 ± 33.24	52.99 ± 39.04	0.784	0.961
*Actinobacteria*	18.0 ± 22.15	2.626 ± 3.099	0.011	0.336
*Proteobacteria*	17.33 ± 22.45	24.58 ± 36.44	0.888	0.987
*Bacteroidetes*	8.311 ± 13.59	14.65 ± 20.97	0.504	0.901
*Fusobacteria*	1.79 ± 7.677	0.279 ± 0.587	0.642	0.961
*Cyanobacteria*	0.263 ± 0.669	0.647 ± 1.123	0.258	0.901
*Synergistetes*	0.183 ± 0.585	0.346 ± 0.657	0.185	0.863
*Tenericutes*	0.392 ± 2.522	0.065 ± 0.089	0.873	0.994
*Spirochaetae*	0.178 ± 0.813	1.599 ± 5.493	0.788	0.916
*Verrucomicrobia*	0.281 ± 0.974	0.453 ± 0.907	0.297	0.901
**Genera level**				
*Corynebacterium*	11.9 ± 20.31	0.154 ± 0.203	0.012	0.638
*Lactobacillus*	17.21 ± 30.05	22.47 ± 39.41	0.989	0.999
*Staphylococcus*	8.167 ± 27.7	8.987 ± 15.89	0.017	0.638
*Streptococcus*	5.915 ± 17.67	9.836 ± 20.99	0.478	0.882
*Dolosigranulum*	0.598 ± 2.506	1.166 ± 3.411	0.509	0.881
*Escherichia-Shigella*	3.715 ± 12.6	0.548 ± 1.743	0.707	0.892
*Propionibacterium*	2.235 ± 4. 646	0.213 ± 0.306	0.275	0.881
*Erysipelotrichaceae*	0.833 ± 2.628	0.038 ± 0.077	0.114	0.882
*Moraxella*	0.08 ± 0.459	12.96 ± 30.88	0.555	0.882
*Ralstonia*	1.522 ± 5.119	0.366 ± 0.48	0.983	0.999
*Pseudomonas*	1.552 ± 10.86	0.067 ± 0.134	0.125	0.881
*Hemophilus*	2.509 ± 12.56	0.155 ± 0.355	0.78	0.918
*Faecalibacterium*	0.589 ± 1.775	0.87 ± 1.708	0.55	0.881
*Enterobacter*	2.675 ± 10.43	0.327 ± 0.842	0.604	0.882
*Peptostreptococcus*	0.34 ± 1.679	0.409 ± 1.319	0.954	0.992
*Candidatus Arthromitus*	0.956 ± 7.197	1.13 ± 2.467	0.299	0.881
*fusobacterium*	1.197 ± 5.897	0.274 ± 0.589	0.751	0.914
*Porphyromonas*	1.592 ± 6.97	0.47 ± 1.604	0.869	0.961
*Rhodococcus*	1.501 ± 3.486	0.658 ± 1.205	0.921	0.981
*Prevotella*	0.474 ± 2.409	4.008 ± 13.69	0.496	0.881
*Bacteroides*	1.906 ± 4.836	4.016 ± 7.451	0.538	0.882
*Neisseria*	0.209 ± 1.191	7.416 ± 25.63	0.721	0.907

At the genus level, we listed the dominant bacteria in the two groups as follows: The dominant bacteria in the non-recurrent group were categorized according to relative abundance: *Lactobacillus* (17.21%), *Corynebacterium* (11.9%), *Staphylococcus* (8.167%), *Dolosigranulum* (7.38%), *Moraxella* (5.44%), *Streptococcus* (5.915%), *Escherichia-Shigella* (3.715%), *Enterobacter* (2.675%), *Hemophilus* (2.509%), *Propionibacterium* (2.235%), *Bacteroides* (1.906%), *Pseudomonades* (1.552%), *Escherichia-Shigella* (2.98%, *Ralstonia* (1.522%), Ralstonia (1.42%) and *Pseudomonades* (1.38%). In contrast, the dominant bacteria in the relapsing group included *Lactobacillus* (22.47%), *Moraxella* (12.96%), *Streptococcus* (9.836%), *Staphylococcus* (8.987%), *Neisseria* (7.416%), *Bacteroides* (4.016%), *Prevotella* (4.008%), *Dolosigranulum* (1.166%) and *Candidatus Arthromitus* (1.13%) (Table 3).

**Figure 4:**
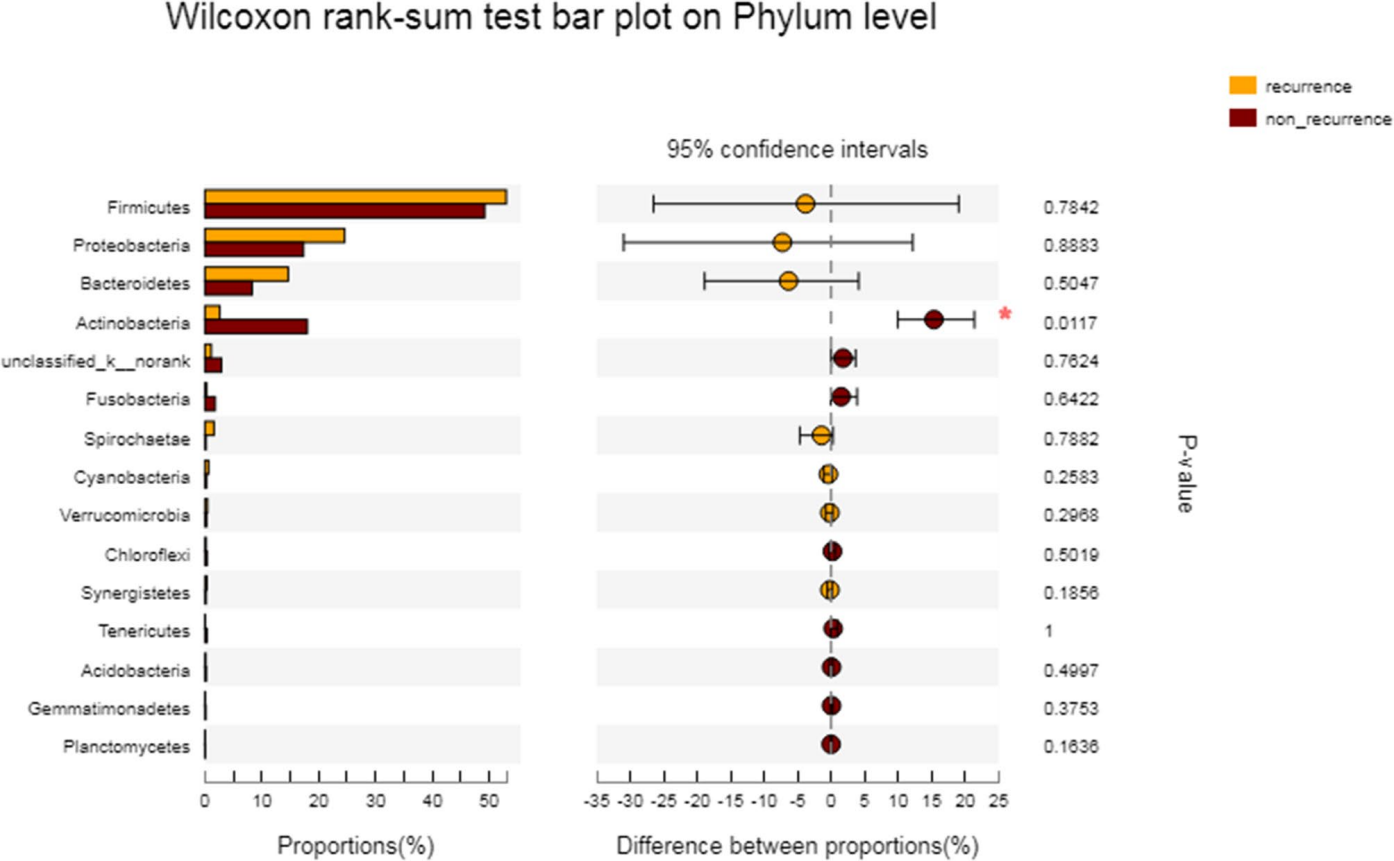
The Wilcoxon rank sum test was used to compare the difference in nasal microbial colony composition between the recurrent and non-recurrent nasal polyp patients at the phylum level.

**Figure 5:**
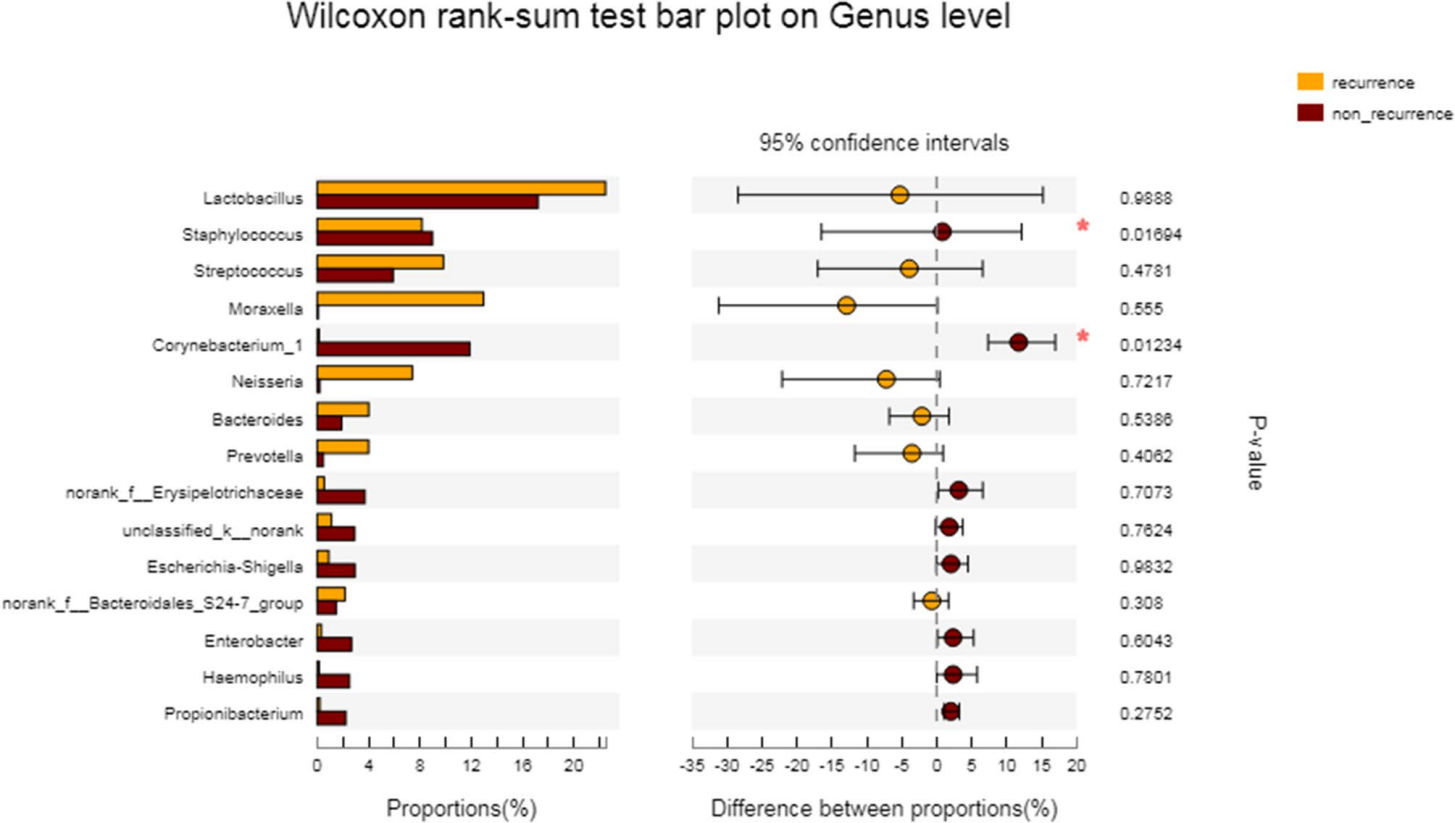
The Wilcoxon rank sum test was used to compare the difference in nasal microbial colony composition between recurrent and non-recurrent nasal polyp patients at the general level.

The relative abundance of bacterial groups recorded in our study is shown in Table 3. Welch's T test was used to compare the differences in nasal microbiome diversity between the two groups at the phylum level and the genus level. At the phylum level, the relative abundance of *Actinobacteria* in the non-recurrent group was 18.0%, while that in the recurrent group was 2.626%. Initially, the difference between the two groups was statistically significant (*P* = 0.011), but after testing and correction, the difference was not statistically significant (RDF *P* > 0.05) (Fig. 4). At the genus level, the relative abundance of *Corynebacterium* was 11.9% in the non-recurrent group, which was significantly higher than that of the recurrent group (0.154%), but the difference was not statistically significant (RDF *P* = 0.638). The relative abundance of *Staphylococcus* in the non-recurrent group was 8.167%, which was significantly lower than that in the recurrent group (8.987%), but the difference was not statistically significant (RDF *P* = 0.638) (Table 3, Fig. 5).

## Discussion

There are some differences in ESS postoperative nasal polyp recurrence rates among many countries around the world. For example, the DeConde team from the United States found that the recurrence rates of CRSwNP patients undergoing ESS surgery were 35%, 38%, and 40% at 6, 12, and 18 months, respectively^8^. Wynn's team, also from the United States, found that up to 40% of patients with nasal polyps had recurrence 40 months after ESS^9^. The Gevaert team from Belgium followed up a European nasal polyp population after ESS for 12 years and found that 80% of the patients had recurrent nasal polyps, and 37% of the patients had to undergo ESS surgery again^10^. In Asian countries, the team of Professor Zhang from Beijing Tongren Hospital found that nasal polyps recurred in up to 55.3% of CRSwNP patients 34 months after surgery, and the recurrence rate of CRSwNP in Southwest China was slightly lower than that in northeast China, presenting an obvious regional difference^11^. The Nakayama team from Japan conducted a 17.5-month follow-up of CRSwNP patients and found that 22.9% of patients with nasal polyps had recurrence^12^. The study in Southwest China in the West China Hospital of Sichuan University of ESS surgery patients with CRSwNP with postoperative follow-up of 1 year found that the nasal polyp recurrence rate was 15.6%; therefore, the postoperative nasal polyp recurrence rate of this region is significantly lower than that of European and American countries and slightly closer to the Japanese team’s findings, but as time goes on, the recurrence rate is likely to rise gradually, so we need to continue to track and conduct a longer long-term follow-up.

In this study, beta diversity of microbial community distribution between CRSwNP patients and the control subjects was calculated by PCoA. We found that most microbial distributions are overlapping and only a minority of samples are specific to each group. At phylum and genus level, some microbial flora in non-CRS group were higher than that in CRSwNP group, mainly concentrated in *Actinobacteria*, *Corynebacterium* and *Dolosigranulum.* After 1-year follow-up, nasal polyp recurrence occurred in 12 patients, including 5 males and 7 females; 3 were asthma patients, 4 patients had allergic rhinitis, and 0 had eczema; there were 8 patients with asthma, 10 patients with allergic rhinitis, and 6 cases with eczema in the CRSwNP non-recurrent group. There was no significant differences between the groups in proportion for age, gender, or allergic diseases such as asthma or allergic rhinitis. In a 40-month follow-up, Wynn's team found that CRSwNP patients with asthma were more prone to nasal polyp recurrence after ESS^9^. Therefore, a longer postoperative follow-up is needed to observe whether the accompanying allergic disease will increase the tendency of nasal polyp recurrence.

In terms of the Lund-Mackay scores and TNSS, the recurrent group was significantly higher than the non-recurrent group, suggesting that patients with severe nasal symptoms or higher Lund-Mackay scores before surgery may have a higher degree of disease severity. After the same ESS treatment, these patients may have a higher risk of nasal polyp recurrence than patients with higher Lund-Mackay scores or lower TNSSs, identical with some previous studies^13–15^. Based on the symptom scores of nasal inflammatory diseases and the distribution characteristics of nasal microorganisms, Copeland found that *E. coli* was positively correlated with the nasal symptom scores of CRS patients^16^. *Enterobacter pylori* is involved in IL-8-mediated neutrophilic inflammation and has a certain correlation in the pathogenesis of nasal polyps. In our study, *Faecalibaculum* was the only bacterium that was negatively correlated with the overall nasal symptoms of CRSwNP patients. It is an obligate anaerobic bacterium that has strong lactic acid fermentation ability and might become a new probiotic to replace *Lactobacillus* and *Bifidobacteria*. The two genera have opposite functions and may have antagonistic effects in the nasal cavity. Due to the influence of the sample quantity and the proportion of grouping, the current study haven’t found that *E. coli* and *Faecalibaculum* differed between the recurrent group and the non-recurrent group, and because of this phenomenon, we can consider expanding the sample size of patients with CRSwNP and having a longer follow-up period, as far as possible further observation, and summarize surgical prognosis if possible correlation with the microflora.

From the relationship between inflammatory cells and nasal polyp recurrence, the degree of eosinophil infiltration in the recurrent group was higher than that in the non-recurrent group, and there was no difference in eosinophil count in peripheral blood between the two groups. Prior study of nasal polyps in the organization degree of eosinophil infiltration^17^ has no clear correlation with postoperative recurrence, and in recent years, research has shown that CRSwNP patients with a higher degree of eosinophil infiltration were characterized by more severe clinical symptoms and a higher recurrence rate, which is consistent with our research conclusion^18,19^. At the same time, we compared the neutrophil count of patients with CRSwNP in peripheral blood and polyp tissue and found that the neutrophil count of the recurrent group was significantly higher than that of the non-recurrent group, which means that the neutrophil-mediated inflammation pattern plays an important role in patients with CRSwNP in Southwest China and is correlated to postoperative recurrence of nasal polyps to some extent.

For inflammatory mediators, the median expression levels of IFN-γ, IL-8, IL-17A, IL-17E and IL-18 were significantly higher in the recurrent group than in the non-recurrent group, while the positive rates of all inflammatory factors, including IL-5 and IL-27, were not significantly different between the recurrent group and the non-recurrent group. IL-8 is known to be a major cytokine produced by activated neutrophils and is positively correlated with the severity of respiratory disease. Derycke et al.^20^ proved that IL-17A can effectively prolong the survival rate of neutrophils in nasal polyp tissue and reduce its apoptosis rate. IFN-γ and IL-18 are activated products and stimulating factors of macrophages, which are closely correlated with anti-inflammatory activities. Therefore, the expression levels of the above cytokines were higher in the recurrent group than in the non-recurrent group, indicating that in this study, the neutrophils and phagocytes in the recurrent group showed a higher degree of activation, mediated more severe respiratory inflammation, and presented an increased risk of postoperative nasal polyp recurrence.

A small percentage of the ESS microbiota remained stable for a long time among the CRSwNP patients. Their stability is mainly reflected in the limited species of dominant bacteria and the large number of low-abundance bacteria. In general, they vary greatly in relative abundance, which constitutes the concept of an individual-specific core sinus microbiome. Although its relative abundance fluctuates among different populations and individuals, with the development of precision medicine, this concept has greatly enriched the hypothesis of the role of bacteria in the pathological mechanism of CRS.

Some researchers found that the nasal bacterial community showed unstable changes after ESS, and the nasal bacterial abundance increased after ESS. The average relative abundance and distribution of many taxa varied, among which *Staphylococcus* was the only dominant taxon whose relative abundance increased significantly. Some experts found that the relative abundance of a rare strain (*Finegoldia magna*) decreased after ESS, the number of patients with higher nasal symptom scores increased, and the number of patients with reduced overall bacterial load decreased. They argues that even with changes in nasal microbial abundance and colony composition after surgery, these data do not support the notion that the prognosis of patients after ESS may be directly attributable to changes in microbial distribution composition^21^. Based on this view, we believe that the diversity of microbial colonies is different in the different follow-up stages, and relatively stable colony structure may also be found in a particular period of time. We monitoring the next follow-up to verify the above idea.

From the perspective of nasal microbiome composition, we found that for both the recurrent and non-recurrent CRSwNP patients in our study, the phyla with higher abundance were all concentrated in the following groups: *Firmicutes*, *Proteobacteria*, *Actinomycetes* and *Bacteroidetes*. The dominant bacteria gerena mainly included *Lactobacillus*, *Staphylococcus*, *Streptococcus* and *Bacteroides*, which was consistent with the previous research results of our team^22^, indicating that the nasal microbial colony composition of CRSwNP patients in a certain region was relatively stable. However, in terms of the nasal microbial richness of the two groups, we found that there was no statistically significant difference in the Sobs index between the recurrent group and the non-recurrent group, indicating that there was no significant difference in the nasal microbiome richness between the two groups before surgery.

In this study, we compared the nasal microbial community structure differences between the CRSwNP population in the recurrent group and the non-recurrent group at both the phylum level and the genera level, and the results showed that there were no statistically significant differences among all the microbial communities. Although the relative abundance of *Actinomycetes* and *Corynebacterium* in the non-recurrent group was significantly higher than that in the recurrent group, the difference was not statistically significant; the relative abundance of *Staphylococci* in the non-recurrent group was significantly lower than that in the recurrent group, and the difference was also not statistically significant, which was consistent with the research results of Jain's team^18^. *Corynebacterium* in *Actinomycetes* phylum is a kind of bacteria that has a protective function in the upper respiratory tract. Hoggard et al.^23^ observed that the bacterial distribution in patients with CRS was different from that in healthy people, mainly manifested as the reduced content of *Corynebacterium*, the dominant strain, in CRS patients compared with healthy adults. Abreu^3^ found that *Corynebacterium* was relatively abundant in the nasal cavity of CRS patients, and the severity of nasal symptoms of these patients (scored using the SNOT 20 scale) was positively correlated with the abundance of *Corynebacterium*. *Staphylococcus aureus* is considered the pathogenic bacterium of sinusitis from the perspective of in vitro culture and high-throughput sequencing, and it mainly induces chronic inflammation of the nasal and sinus mucosa through bacterial surface antigens^24,25^. Uehara^26^ found a relationship between survival competition and inhibition between intranasal *S. aureus* and *Corynebacterium*. The incidence of *S. aureus* colonization in Corynebacter nasal carriers (8.5%) was significantly lower than that in noncarriers (44.5%). In addition, when the Corynebacterium strain was artificially implanted into the nasal cavity of a *S. aureus* carrier, approximately 71% of the subjects had *S. aureus* removed from the nose.

In our study, we observed a decrease in *Corynebacterium* and an increase in *Staphylococci* in the recurrent group of CRSwNP patients, which means that in the recurrent group, the abundance of bacteria with a protective function is relatively lower, while the abundance of *S. aureus* with pathogenic effects is relatively higher, which is an important factor in the nasal microbiome composition in the recurrence of nasal polyps. We will further analyze the differences in nasal microbial composition between the two groups by increasing the sample size and extending the follow-up time, hoping to obtain more information about the correlation between nasal microbiome diversity and CRSwNP recurrence.

We found that postoperative recurrence of nasal polyps was not associated with age, gender, asthma, allergic rhinitis or other allergic diseases in CRSwNP patients. From the TNSSs, patients with more severe nasal symptoms before surgery may have a higher risk of nasal polyp recurrence. The neutrophil-mediated inflammatory response plays an important role in patients with CRSwNP in Southwest China and may be associated with nasal polyp recurrence. In the recurrent group, the abundance of protective bacteria was relatively lower, while the abundance of pathogenic *S. aureus* was relatively higher, which is an important factor in nasal microbiome composition for nasal polyp recurrence.

## Materials and methods

### Subjects

A total of 77 CRSwNP patients who underwent endoscopic sinus surgery (ESS) by the author (Liu F.) between December 2017 and December 2018 were recruited into the study at the Department of Otorhinolaryngology of the West China Hospital, Sichuan University, Chengdu, Sichuan. The surgery mainly involves removal of polyps from the nasal cavity, removal of inflamed or dysfunctional mucosa, conservation of normal mucosa and sinus open. All patients were advised to regularly use intranasal hormones and saline nasal irrigation after surgery. Follow-up was performed at least 1 month, 3 months, 6 months and 1 year after surgery.

The diagnosis of CRSwNP or CRSsNP was based on patient history, clinical examination, nasal endoscopy and computed tomography results according to the current EPOS guidelines^27^. During the follow-up after ESS and medication, if objective CT indicated abnormal density shadow in nasal meatus or sinuses, and endoscopy showed mucosal thickening, mucinous purulent discharge, mucosal edema, or polypoid changes, recurrence was considered. Patients who had received antibiotics and systemic or topical intranasal steroid treatments within 4 weeks before the operation were excluded. This time frame was based on a previous study showing that the gut microbiome returned to a steady level within 4 weeks after the cessation of antibiotic use^28^. Moreover, patients with cystic fibrosis, an immunocompromised status or autoimmune disease, immunodeficiency, pregnancy or a primary mucociliary impairment and those less than 18 years old were excluded from the study.

Patients who underwent ESS surgery and had no sinus inflammation diseases, including nasal septum deviation, inverted papilloma, pituitary adenomas, chronic dacryocystitis, or optical canal fractures, were recruited as the control group.

Clinical data of CRSwNP patients were collected, including age, gender, smoking history, alcohol consumption history, body mass index (BMI), allergic disease history, total clinical symptom score (TNSS), Lund-Mackay score, and Davos score.

The study was approved by the ethics committee of West China Hospital, Sichuan University. All patients gave their written informed consent before participation.

### Sample collection

Following the induction of general anaesthesia, sampling was immediately performed prior to the removal of nasal secretions and the application of topical mucosal vasoconstrictors. Sterile swabs (Gongdong Medical Technology, Taizhou, Zhejiang, CN) were endoscopically guided to the middle meatus region, rotated for 5 full turns and kept in place for at least one minute until fully saturated. Care was taken to avoid contamination from the anterior nasal cavity during swabbing. Any swabs that were contaminated through contact with a nontarget region were discarded. The collected samples were placed in 2-ml sterile Corning freezing tubes without enzyme on ice temporarily, transported to the laboratory within 2 h and stored at − 80 °C in liquid nitrogen until DNA extraction was performed^22,29^.

Nasal polyp tissues were collected, stored in two parts: one was fixed with paraformaldehyde for HE and immunohistochemical staining, and the other was frozen at − 80 °C and homogenized to detect the expression of IL-5, IL-8, IL-17A, IL-17E, IL-18, IL-27 and IFN-γ. Blood samples were obtained to count inflammatory cells by flow cytometer.

During the 1-year postoperative follow-up, symptoms were recorded and nasal endoscopy and sinus CT examination were performed to determine whether nasal polyps had recurred, and middle nasal meatus secretions were collected at the same time.

### Measurement of inflammatory factors

Samples for inflammatory factor assessment using immunoassays were weighed. A total of 1.0 ml of 0.9% NaCl was added per 0.1 g of each sample, and all samples were homogenized at 1000 rpm for 5 min and centrifuged at 1500 g for 10 min at 4 °C. Supernatants were collected and stored at − 20 °C until analysis. IL-5, IL-8, IL-17A, IL-17E, IL-18, IL-27 and IFN-γ concentrations were assessed using a Luminex 100 system (Luminex, Austin, TX, USA).

### Immunohistochemical staining

Streptomyces antibiotinin protein-peroxidase conjugating method (SP method) was performed by eosinophilic cationic protein (ECP) and myeloperoxidase (MPO). The brownish cells in the cytoplasm of various inflammatory cells are positive cells. 10 fields of 400-fold light microscopy were randomly selected in each sample. A manual counter was used to count the number of positive cells, and then the average value of the 10 counts was used to count the positive inflammatory cells in the samples. The two researchers made the decision double-blind.

### Microbial genomic DNA extraction

Microbial DNA was extracted from the swab samples using the DNA Kit (Omega Bio-tek, Norcross, GA, USA) according to the manufacturer’s protocols. The V3-V4 region of the bacteria’s 16S ribosomal RNA gene were amplified by PCR (95 °C for 3 min, followed by 27 cycles at 95 °C for 30 s, 55 °C for 30 s, 72 °C for 45 s and a final extension at 72 °C for 10 min) using the primers 338F 5′-barcode-ACTCCTAGGGAGGCAGCAG)-3′ and 806R 5′-GGACTACHVGGGTWTCTAAT-3′, where the barcode is an eight-base sequence unique to each sample. PCR reactions were performed in a triplicate 20 μL mixture containing 4 μL of 5 × FastPfu Buffer, 2 μL of 2.5 mM dNTPs, 0.8 μL of each primer (5 μM), 0.4 μL of FastPfu Polymerase, and 10 ng of template DNA^22,29^.

### Illumina MiSeq sequencing

Illumina MiSeq is a next-generation sequencing (NGS) method in which high-quality 16S rRNA sequences are generated for the subject. Amplicons were extracted from 2% agarose gels, purified using the AxyPrep DNA Gel Extraction Kit (Axygen Biosciences, Union City, CA, USA) according to the manufacturer’s instructions and quantified using QuantiFluor-ST (Promega, USA). The purified amplicons were pooled in equimolar amounts and paired-end sequenced (2 × 250) on the Illumina MiSeq platform according to standard protocols. V3–V4 variable regions of the bacterial 16S rRNA gene were generated for the amplicons^22,29^.

### Statistical analysis

Demographic and clinical characteristics were analysed using the chi-square test for binominal variables and the Mann–Whitney U test for continuous variables, such as Lund-Mackay score and visual analogue scale (VAS) score. We used Welch’s t-test to analyse the microbiota richness index between groups. We used the UPARSE calculation in the clustering process, which excluded single-read OTUs automatically. Alpha diversity indices were calculated for all samples at 97% OTU similarity using mothur index analysis. Alpha diversity indices include the Sobs, Shannon, and Simpson indices. The Sobs index was calculated to reflect community richness, and the Shannon and Simpson indices were calculated to determine community diversity. Principal coordinate analysis (PCoA) was used to explore and visualize data similarities in three-dimensional space. Distance matrices were calculated using the Morisita-Horn index (vegdist function of the R package vegan) and applied to the cmdscale function in R. The differences in the relative abundance between the two groups were evaluated using the Wilcoxon rank sum test with the false discovery rate (FDR) multiple testing correction. When *P* < 0.05 (2-sided), we considered the difference significant.

All methods were carried out in accordance with relevant guidelines and regulations.

### Ethics approval and consent to participate

The study was approved by the ethics committee of West China Hospital, Sichuan University (NO. 2017-448). All patients gave their written informed consent before participation.

### Consent to publish

All authors consent to publish this manuscript to Scientific Reports.

## Data Availability

The datasets generated and analysed during the current study are available in the National Center for Biotechnology Information (NCBI) Sequence Read Archive (SRA) database(Accession Number: SRP162948, ftp://ftp.ncbi.nlm.nih.gov/sra/sra-instant/reads/ByStudy/sra/SRP/SRP162/SRP162948/).

## Abbreviations

CRSwNP: Chronic rhinosinusitis with polyps
TNSS: Total nasal symptom score
ESS: Endoscopic sinus surgery
rRNA: Ribosomal RNA
BMI: Body mass index
SP method: Streptomyces antibiotinin protein-peroxidase conjugating method
ECP: Eosinophilic cationic protein
MPO: Myeloperoxidase
NGS: Next-generation sequencing
NCBI: National Center for Biotechnology Information
SRA: Sequence Read Archive
FLASH: Fast low-angle shot magnetic resonance imaging
OTUs: Operational taxonomic units
VAS: Visual analogue scale
PCoA: Principal coordinate analysis
FDR: False discovery rate
KW: Kruskal–Wallis
LDA: Linear discriminant analysis
